# Anatomic analysis of the whole articular capsule of the shoulder joint, with reference to the capsular attachment and thickness

**DOI:** 10.1186/s40634-018-0134-8

**Published:** 2018-06-07

**Authors:** Daisuke Momma, Akimoto Nimura, Satoru Muro, Hitomi Fujishiro, Takashi Miyamoto, Tadanao Funakoshi, Tomoyuki Mochizuki, Norimasa Iwasaki, Keiichi Akita

**Affiliations:** 1 Department of Orthopaedic Surgery, Graduate School of Medicine, Hokkaido University, Hokkaido, Japan; 2 Department of Functional Joint Anatomy, Graduate School of Medical and Dental Sciences, Tokyo Medical and Dental University, Tokyo, Japan; 3 Department of Clinical Anatomy, Graduate School of Medical and Dental Sciences, Tokyo Medical and Dental University, Tokyo, Japan; 4 Department of Orthopaedic Surgery, Nissan Tamagawa Hospital, Tokyo, Japan

## Abstract

**Background:**

Although conventional Bankart repair has been the accepted procedure for traumatic anterior glenohumeral instability, the humeral avulsion of the glenohumeral ligament or an elongation of the capsule remains challenging to decide the appropriate treatment. The anatomical knowledge regarding the whole capsule of glenohumeral joint is necessary to accurately treat for the capsular disorders. The aims of the current study were to investigate the anatomical features of capsular attachment and thickness in a whole capsule of glenohumeral joint.

**Methods:**

We used 13 shoulders in the current study. In 9 shoulders, we macroscopically measured the attachment widths of the capsulolabrum complex on the scapular glenoid, and the attachment widths of the capsule on the humerus in reference to the scapular origin of the long head of triceps brachii, and the humeral insertion of the rotator cuff tendons. We additionally used 4 cadaveric shoulders, which were embalmed using Thiel’s method, for the analysis of the thickness in a whole capsule by using micro-CT.

**Results:**

The glenoidal attachment of the articular capsule appeared to have a consistent width except for the superior part of the origin of the long head of triceps brachii. On the humerus, the articular capsule was widely attached to areas without overlying rotator cuffs, with the widest width (17.3 ± 0.9 mm) attached to the axillary pouch. The inferior part of the capsule, which was consistently thicker than the superior part, continued to the superior part along the glenoid and humeral side edge.

**Conclusions:**

The current study showed that the inferior part of the glenohumeral capsule had a wide humeral attachment from the inferior edge of the subscapularis insertion to the inferior edge of the teres minor insertion via the anatomical neck of the humerus, and the thickness of it was thicker than the superior part of the capsule.

## Background

Several biomechanical and clinical studies have shown that labral detachment from the glenoid and elongation of the articular capsule are essential lesions for the recurrent anterior glenohumeral instability, known as, Bankart lesions.(Bankart 1938; Bigliani et al. 1992; Boileau et al. 2006; Hayashida et al. 1998; Speer et al. 1994) Arthroscopic or open Bankart repair has been recognized as a gold standard for traumatic anterior glenohumeral instability.(Gill et al. 1997; Rowe et al. 1978) In contrast, the treatment for glenohumeral capsule disorder, such as the humeral avulsion of the glenohumeral ligament or an elongation of the capsule, remains challenging to decide the appropriate surgical techniques.

Based on dissection studies, Nimura et al.(Nimura et al. 2012) showed the wide attachment of the articular capsule at the border between the infraspinatus and the teres minor. Moreover, the attachment of the posterosuperior capsule may contribute to the stability of the glenohumeral joint.(Nimura and Akita 2013) Recently, it has been though that anatomical knowledge regarding the relatively wide attachment of the joint capsule could be helpful for the understanding the joint stabilizing in other joints.(Nasu et al. 2017; Nimura et al. 2014; Sato et al. 2018; Shimura et al. 2016) However, the anterior and inferior attachments of the articular capsule, which is generally referred as the inferior glenohumeral ligament, on the humerus has been remained controversial. Furthermore, the relationship between the width of the capsular attachment and the thickness of the corresponding midsubstance of the capsule has not been assessed. If the wide attachment of the capsule were shown to correspond to the thick capsule in the whole capsule, the anatomical significance of the wide attachment of the capsule could be understood and useful for the selection of the operative procedures.

Our objectives were as follows: 1) to measure the width of the capsular attachments on both the humerus and glenoid edge of the scapula, and 2) to comprehensively analyze the thickness measurements of the whole capsule in association with the corresponding attachments on bones. We hypothesize that the attachment widths of the shoulder joint capsule vary in association with the bony location, and correlate with the local thickness of the whole capsule.

## Methods

### Donors and embalming methods

A total of 14 shoulders from 11 Japanese cadavers (4 males and 7 females; average age, 82.8 years old, range, 59 to 98 years old) were included in this study. All cadavers were donated to Tokyo Medical and Dental University. All of the donors had voluntarily declared that their remains were to be donated as materials for education and study. This voluntary donor system of cadavers is in place throughout Japan, and our study completely complies with the current Japanese laws. Of 14 shoulders, 10 shoulders were embalmed in 8% formalin and preserved in 30% ethanol and used for only macroscopic observations and measurements of attachment widths. In addition, of 14 shoulders, the remaining 4 cadaveric shoulders (average age, 84.0 years old, range, 78 to 90 years old) were embalmed using Thiel’s embalming method(Thiel 2002) for the purpose of analysis of capsular thickness. We used the Thiel’s embalming cadavers to eliminate the possibility of the bias associated with the shoulder position at the time of embalming. Thiel’s embalming method can preserve the life-like tissue qualities and flexibility of joints for long term without the need for refrigeration which compromises tissue quality.(Eisma et al. 2013; Healy et al. 2015) The embalming fluids in this method are water based and contain boric acid, ammonium nitrate, potassium nitrate, sodium sulphite, propylene glycol, formaldehyde solution, morpholine, and alcohol.

### Measurements for widths of the area of capsular attachments

**Fig. 1:**
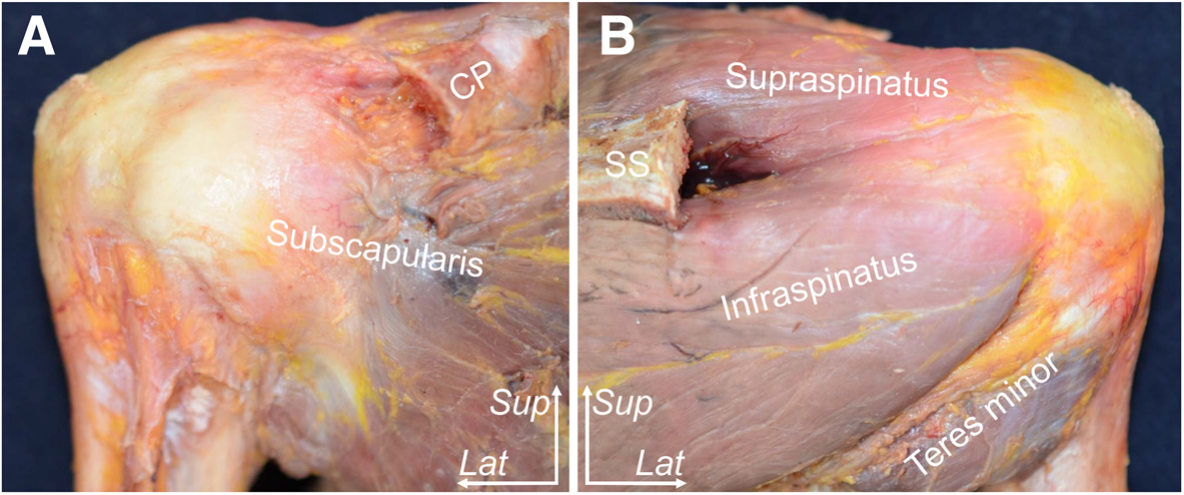
Muscular and tendinous structures of the subscapularis, supraspinatus, infraspinatus, and teres minor near their insertions into the humerus (right shoulder, the acromion and coracoid process has been removed). **a** Anterior aspect of the shoulder. **b** Posterior aspect of the shoulder. CP, coracoid process; SS, scapula spine; *Lat*, lateral; *Sup*, superior

**Fig. 2:**
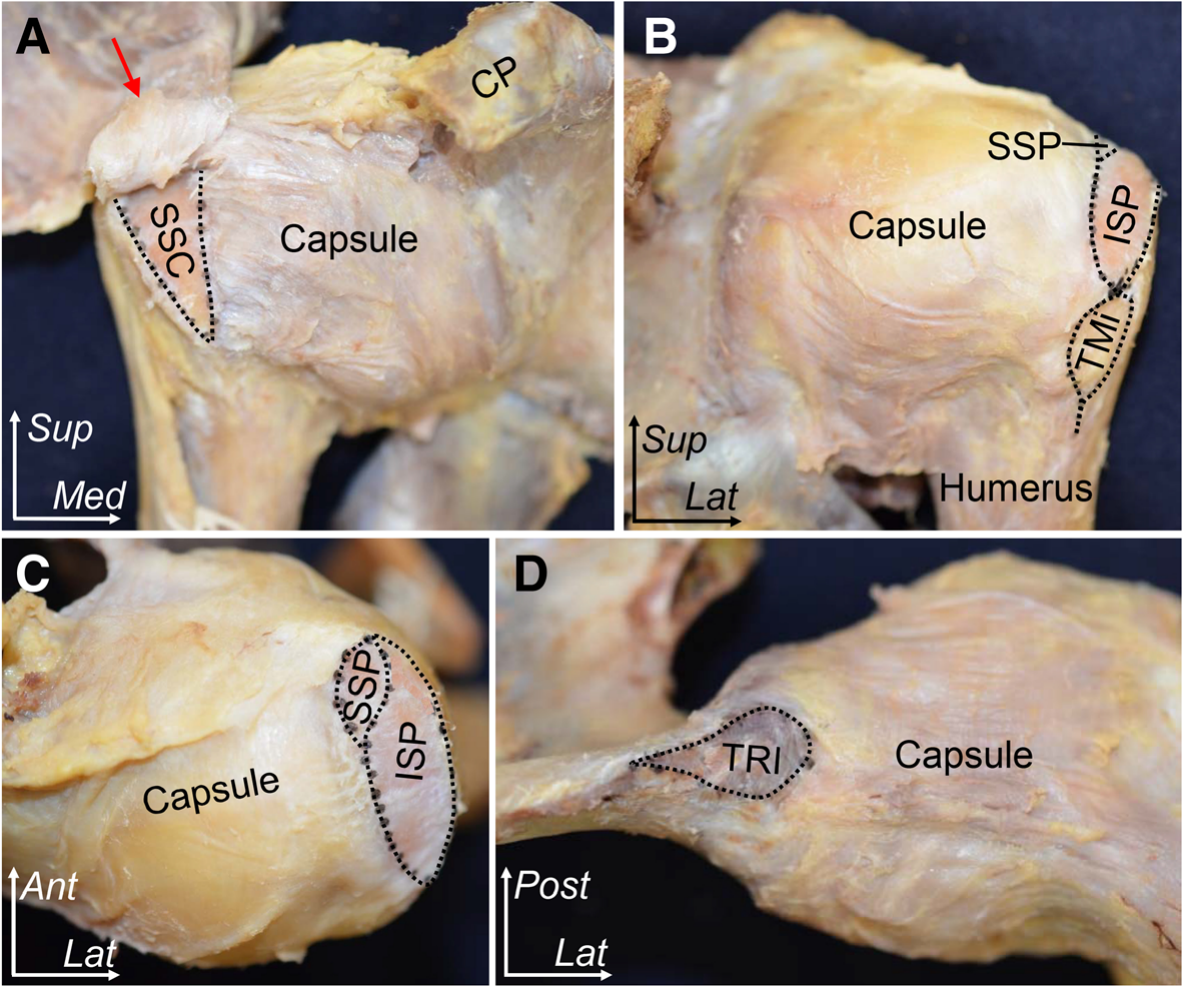
Outward appearance of the shoulder joint. Areas outlined by black dotted lines indicate insertions of the subscapularis (SSC), supraspinatus (SSP), infraspinatus (ISP), teres minor (TMi), and the origin of long head of triceps brachii (TRI). **a** Anterior aspect of the shoulder. The superior-most part of the subscapularis was left intact because it was tightly connected to the capsule and the coracohumeral ligament (red arrow). **b** Posterior aspect. **c** Superior aspect. **d** Inferior aspect. CP, coracoid process; SS, scapula spine; TRI, triceps; *Ant*, anterior; *Med*, medial; *Lat*, lateral; *Sup*, superior; *Post*, posterior

**Fig. 3:**
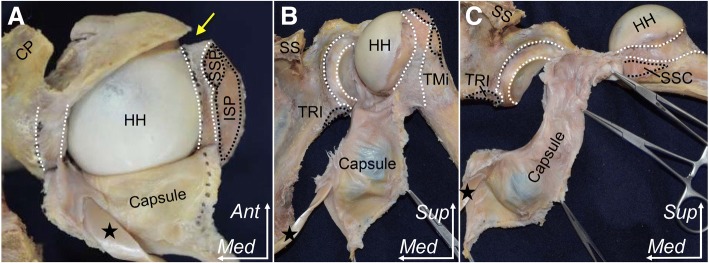
Detaching the articular capsule from the right shoulder joint. The rotator interval was opened at the anterior edge of the supraspinatus muscle, which is the same site as the lateral edge of the intertubercular groove (yellow arrow). The articular capsule including the labrum with the long head of biceps brachii (black star) was detached from the glenoid and the humerus in the following order: superior (**a**), posterior (**b**), inferior and anterior parts of the shoulder joint (**c**). Areas marked with white dotted lines indicate attachments of the articular capsule. CP, coracoid process; HH, humeral head; ISP, insertion of infraspinatus; SSC, insertion of subscapularis; SS, scapular spine; SSP, insertion of supraspinatus; TMi, insertion of teres minor; TRI, origin of long head of triceps brachii; *Ant*, anterior; *Med*, medial; *Sup*, superior

The entire scapula, proximal third of the humerus, and distal half of the clavicle with soft tissues were obtained by cutting the humerus and clavicle. The skin, subcutaneous tissues, and deltoid muscle were removed from the shoulder. After resection of the acromion and the base of coracoid process, the long head of the triceps brachii was removed from the infraglenoidal tubercle and the origin was marked. The supraspinatus, infraspinatus, teres minor, and subscapularis muscles were identified (Fig. 1); after marking the insertions, they were then peeled away from their origin on the scapula to their insertion on the humerus while keeping the glenohumeral capsule intact (Fig. 2). At that step, one shoulder with torn tendon was excluded due to difficulty in precise delineation of the articular capsule attachment. Then, the rotator interval was opened. And the capsule with labrum or biceps labrum complex was detached from the glenoid attachment. The parts of the humeral attachment of the capsule were detached from the humerus in following order: superior, inferior and anterior parts (Fig. 3). Finally, we measured attachment widths of the articular capsule of 9 shoulders (average age, 83.3 years old, range, 59 to 98 years old) on the glenoid and humerus using a caliper (SM7, KANON, Tokyo, Japan). To evaluate the validity of measurement within each group, we measured each value twice on different days and calculated the intraclass correlation coefficient. We used the data firstly measured as the actual result.

### Analysis of the thickness of the whole capsule by micro-CT

Micro-CT was used to evaluate the relationship between attachment width of the articular capsule on the humerus and the distribution of capsular thickness. After detaching from the glenoid and humerus, the whole capsule from 4 cadaveric shoulders which were embalmed using Thiel’s method as described above, including the biceps labrum complex, was analyzed based on 3D measurement using micro computed tomography (micro-CT) for analysis of the local thickness. A high-resolution micro-CT scanner (inspeXio SMX-100CT, Shimadzu Corporation, Kyoto, Japan) was used to obtain articular capsule images. The obtained image data was transferred to the image analysis system, Image J software (version 1.41; National Institutes of Health, Bethesda, MD), for visualization of the thickness distribution. First, binarization for all sequential frames which were extracted from input micro-CT images by specifying a CT value. Second, an image stack is projected along the axis perpendicular to the image plane (the so-called articular capsular thickness). This projection creates a real image that is the sum of the binary slices in the stack. The thickness of the articular capsule was then calculated based on slice unit length and slice number. Finally, color balance was performed to recognize the location specificity of the articular capsule as visulalized by OsiriX (Pixmeo, Benex, Switzerland). The measured thickness at each coordinate point were mapped with a 7-grade color scale, in which red, orange, yello, green, blue, violet, and black lined up in order. Red indicates the maximum thickness (8 mm), and black indicates the minimum one (0.1 mm). The whole capsule was divided into 3 approximate parts attachments on humerus; anterior (*Ant*), inferior (*Inf*), and superior (*Sup*), according to the site of attachment. *Ant* corresponded to the inferior edge of subscapularis insertion, *Inf* corresponded to the most inferior attachment of the capsule, and *Sup* corresponded to the margin between infraspinatus and teres minor insertion. The thicknesses of adjacent parts of the whole capsule were compared based on the visualized color as a data of the thickness.

### Statistical analysis

Statistical analyses were performed by using JMP 12.0.1 (SAS Institute Inc., Cary, NC). Statistical comparisons among the measured locations were conducted with analysis of variance and the Tukey-Kramer post-hoc test. Differences were considered significant at *P* < .05. Interobserver reliabilities were determined with evaluating the measurement process (EMP) analysis.

## Results

### Measurements for widths of the area of capsular attachments

**Fig. 4:**
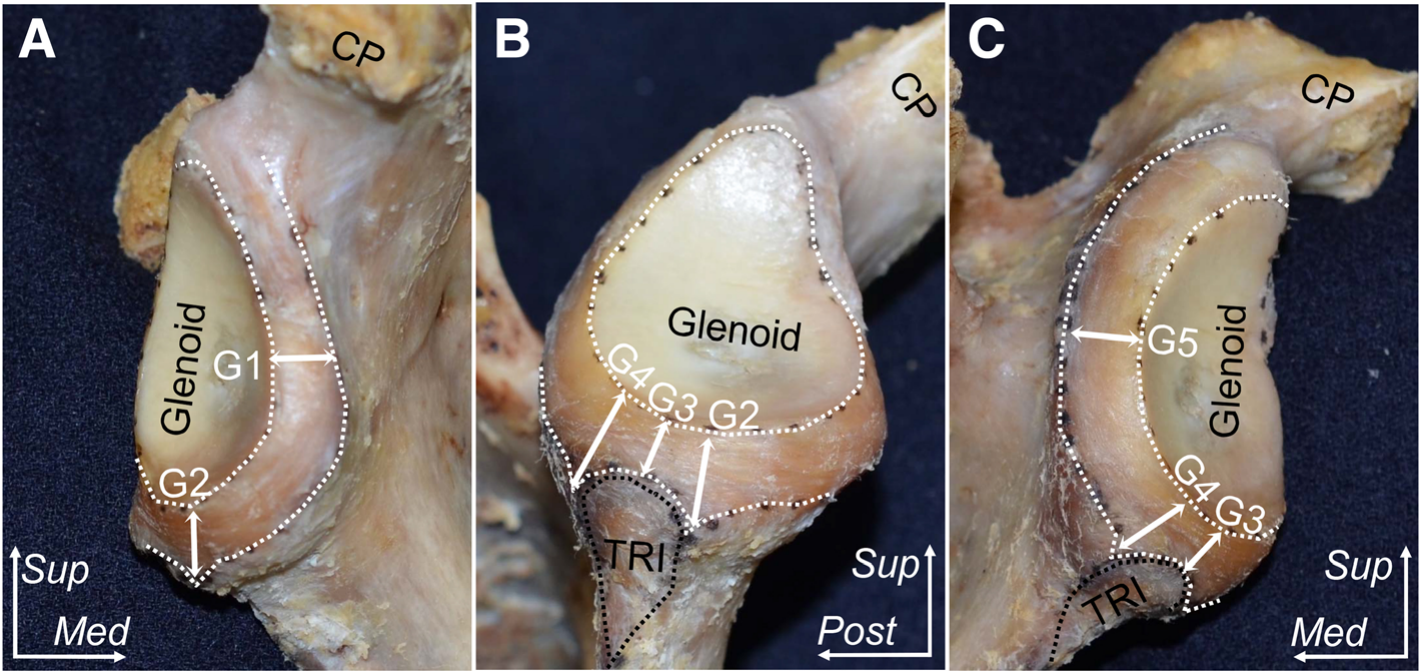
The glenoidal attachment of articular capsule. The area marked with white dotted lines indicates the attachment area of the articular capsule. **a** Anterior aspect of the glenoid. **b** Inferior aspect. **c** Posterior aspect. Attachment widths were measured at 3 o’clock position of the glenoid (G1), the anterior edge (G2), superior part (G3), and posterior edge (G4) of the origin of the long head of triceps brachii, and 9 o’clock position of the glenoid (G5). Measurement data are listed in Table 1. CP, coracoid process; TRI, origin of long head of triceps brachii; *Med*, medial; *Post*, posterior; *Sup*, superior

Regarding the glenoidal attachment of the articular capsule, we could not separate the capsule from the labrum. The widths of the attachment of the capsulolabrum complex appeared consistent around the whole glenoid except for the superior part of the origin of the long head of triceps brachii (Fig. 4). At the anterior and posterior part of the glenoid edge, the average widths (± standard deviation) of the capsular attachment connecting to the labrum were 5.7 ± 0.5, 6.1 ± 0.3 mm, respectively (G1 in Fig. 4a, G5 in Fig. 4c, Table 1). At the anterior and posterior edges of the scapular origin of the long head of triceps brachii, the average widths were 7.6 ± 0.3, 6.0 ± 0.3 mm, respectively (G4 in Fig. 4b, G2 in Fig. 4a, Table 1). Superior to the scapular origin of the long head of triceps brachii, the average width (4.6 ± 0.2 mm, G3 in Fig. 4b, Table 1) was significantly narrower than only G2 (*P* = .0022).

**Table 1:** Measurements of the glenoidal attachment of the articular capsule

Measurement locations	Average width and standard deviation (mm)
Anterior part of the glenoidal edge at 3 o’clock (G1)	5.6 ± 0.5
Anterior edge of origin of long head of triceps brachii (G2)	7.6 ± 0.3
Superior part of origin of long head of triceps brachii (G3)	*4.6 ± 0.2
Posterior edge of origin of long head of triceps brachii (G4)	6.0 ± 0.3
Posterior part of the glenoidal edge at 9 o’clock (G5)	6.1 ± 0.3

Measurement locations are shown in Fig. 4. G3 (asterisk) was significantly narrower than only G2 (*P* = .022 by ANOVA with Tukey-Kramer post-hoc test)

**Fig. 5:**
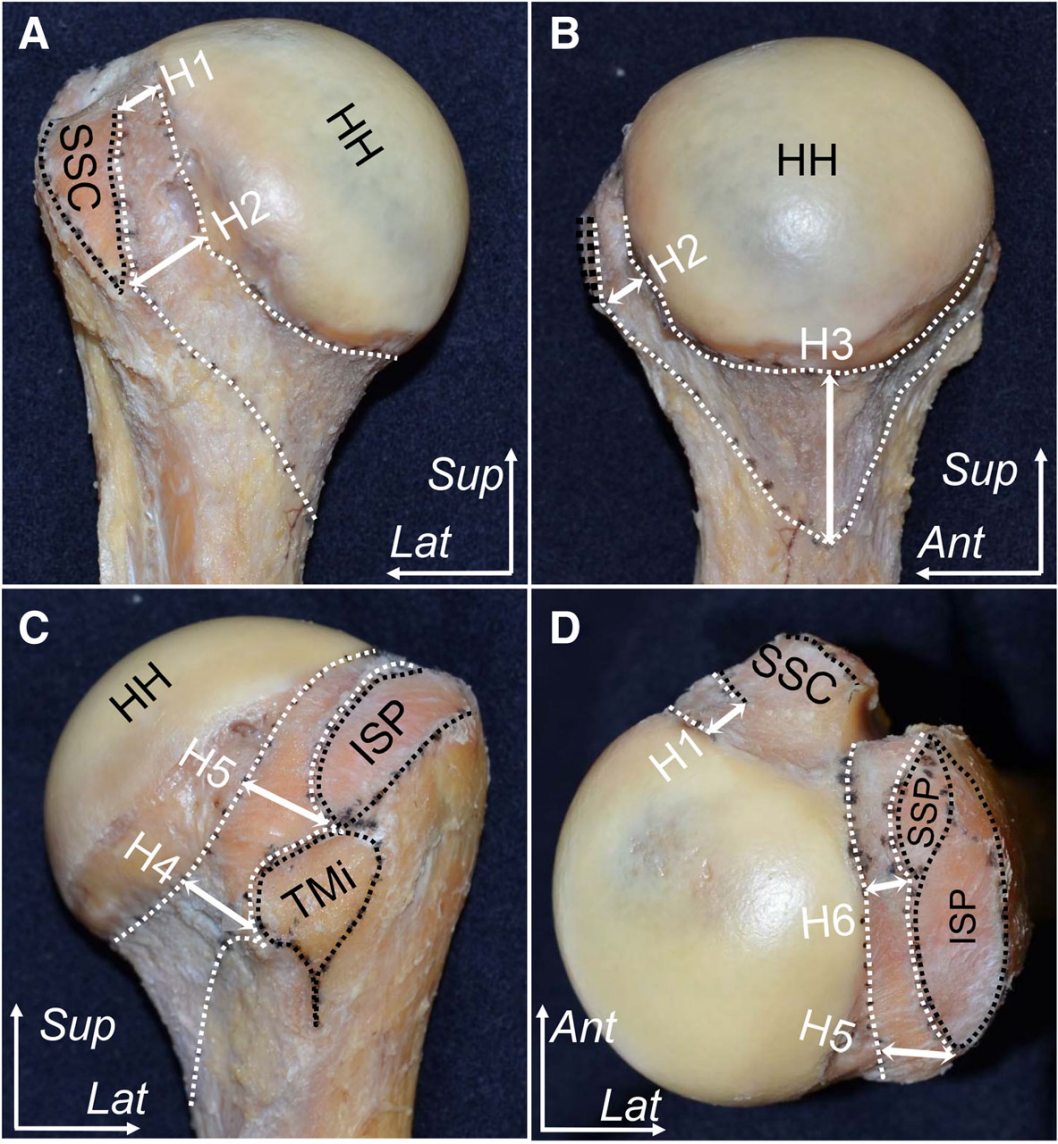
Humeral attachments of the articular capsule. The area marked with white dotted lines indicates the attachment area of the articular capsule. **a** Anterior aspect of the humerus. **b** Medial aspect. **c** Posterior aspect. **d** Superior aspect. Attachment widths were measured at superior edge of the subscapularis insertion (H1), the inferior edge of the subscapularis insertion (H2), the widest attachment at the axillary pouch (H3), the inferior edge of the tendinous insertion of teres minor (H4), the border between the infraspinatus and teres minor insertion (H5), and the posterior edge of the supraspinatus insertion (H6). Measurement data are listed in Table 2. HH, humeral head; ISP, insertion of infraspinatus; SSC, insertion of subscapularis; SSP, insertion of supraspinatus; TMi, insertion of teres minor; *Ant*, anterior; *Lat*, lateral; *Med*, medial; *Post*, posterior; *Sup*, superior

In contrast, the widths of the capsular attachments to the humerus varied according to location. The capsule attached with a relatively narrow width to the site where rotator cuffs were broadly inserted into the humerus. At the superior edge of the subscapularis insertion, in which the tendon and capsule could be separated, the widths of the capsular attachment was 5.1 ± 0.5 mm (H1 in Fig. 5, Table 2). The posterior edge of the supraspinatus insertion, the widths of the capsular attachment was 5.2 ± 0.3 mm (H6 in Fig. 5, Table 2). At the inferior edge of the subscapularis and teres minor insertion, widths of the capsular attachment were 10.7 ± 0.6 mm (H2 in Fig. 5, Table 2), and 8.5 ± 0.5 mm (H4 in Fig. 5, Table 2), respectively. In addition, at the border between the infraspinatus and teres minor insertion, the width of the capsular attachment was 8.3 ± 0.5 (H5 in Fig. 5, Table 2). In particular, the capsule attached to the axillary pouch with the widest width, at the site lacking the insertion of the rotator cuff (17.3 ± 0.9 mm, H3 in Fig. 5, Table 2). There were significant differences compared to others (*P* = .015). Analysis of interobserver reliability regarding the measurement of capsular attachments yielded an intraclass correlation coefficient of 0.97–0.99 with EMP analysis.

**Table 2:** Measurements of the humeral attachment of the articular capsule

Measurement locations	Average width and standard deviation (mm)
Superior edge of subscapularis insertion (H1)	5.1 ± 0.5
Inferior edge of subscapularis insertion (H2)	10.7 ± 0.6
Axillary pouch of shoulder joint (H3)	*17.3 ± 0.9
Inferior edge of teres minor insertion (H4)	8.5 ± 0.5
Border between infraspinatus and teres minor insertion (H5)	8.3 ± 0.5
Posterior edge of supraspinatus insertion (H6)	5.2 ± 0.3

Measurement locations are shown in Fig. 5. Asterisk indicates statistically significant difference compared to others (*P* = .015 by ANOVA with Tukey-Kramer post-hoc test)

### Visualization of the local thickness of the whole glenohumeral capsule using micro computed tomography

**Fig. 6:**
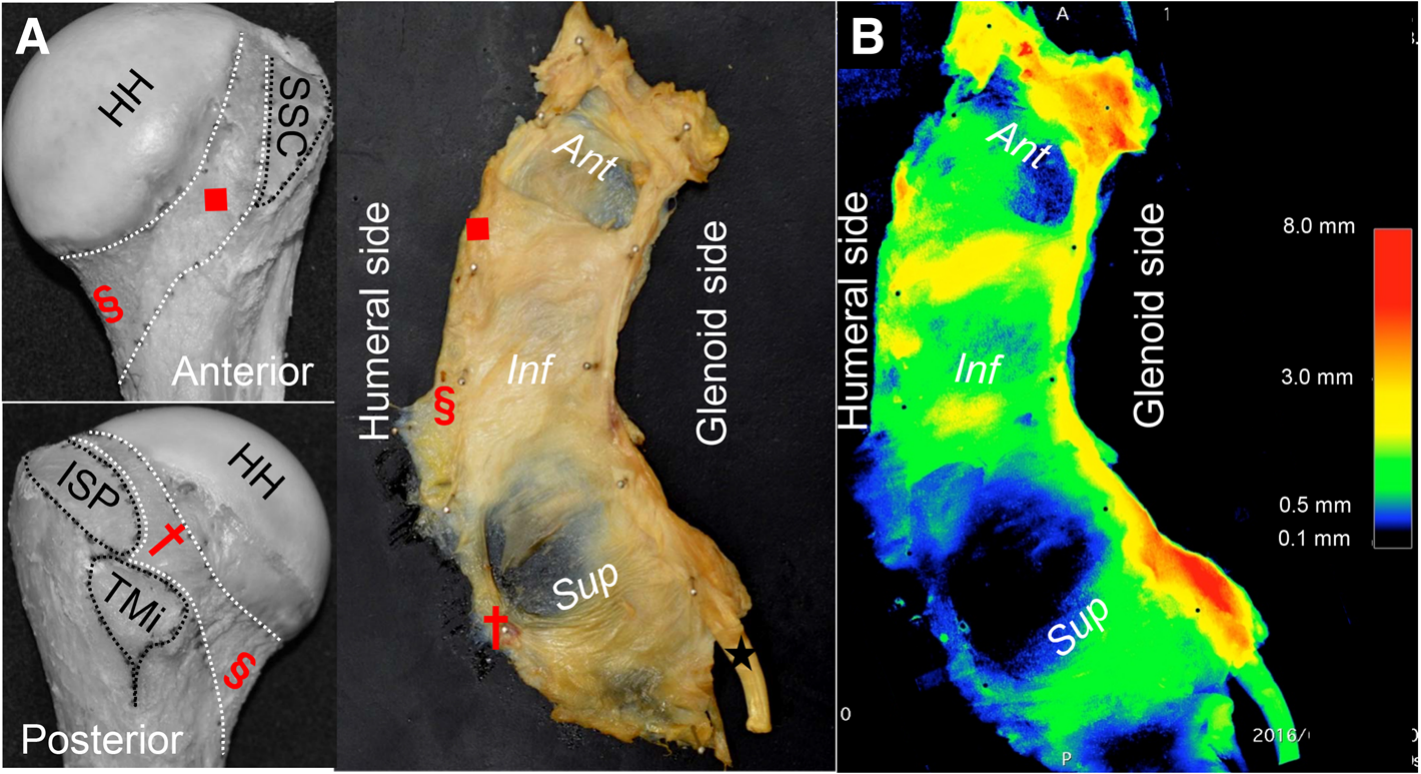
Analysis of the distribution of the thickness in the whole capsule using micro-CT. After detaching from the glenoid and humerus, the whole capsule, including the biceps labrum complex, was analyzed based on 3D measurement using micro-CT. **a** Three landmarks of humeral attachment at the inferior edge of subscapularis insertion (red square), most inferior (red section mark), the margin between infraspinatus and teres minor (red cross) are shown in pictures of the humerus. The inside appearance of the whole capsule, including the long head of biceps brachii (black star), which was flattened is also shown. The whole capsule was divided into 3 approximate parts; anterior (*Ant*), inferior (*Inf*), and superior (*Sup*) according to the site of attachment. Three landmarks correspond to each other. **b** The distribution of thickness on the whole capsule which is shown in (**a**) could be identified in the 3D CT image by micro-CT. The color bar represents the approximate thickness (mm) corresponding to the colors. HH, humeral head; SSC, insertion of subscapularis; TMi, insertion of teres minor; ISP, insertion of infraspinatus

**Fig. 7:**
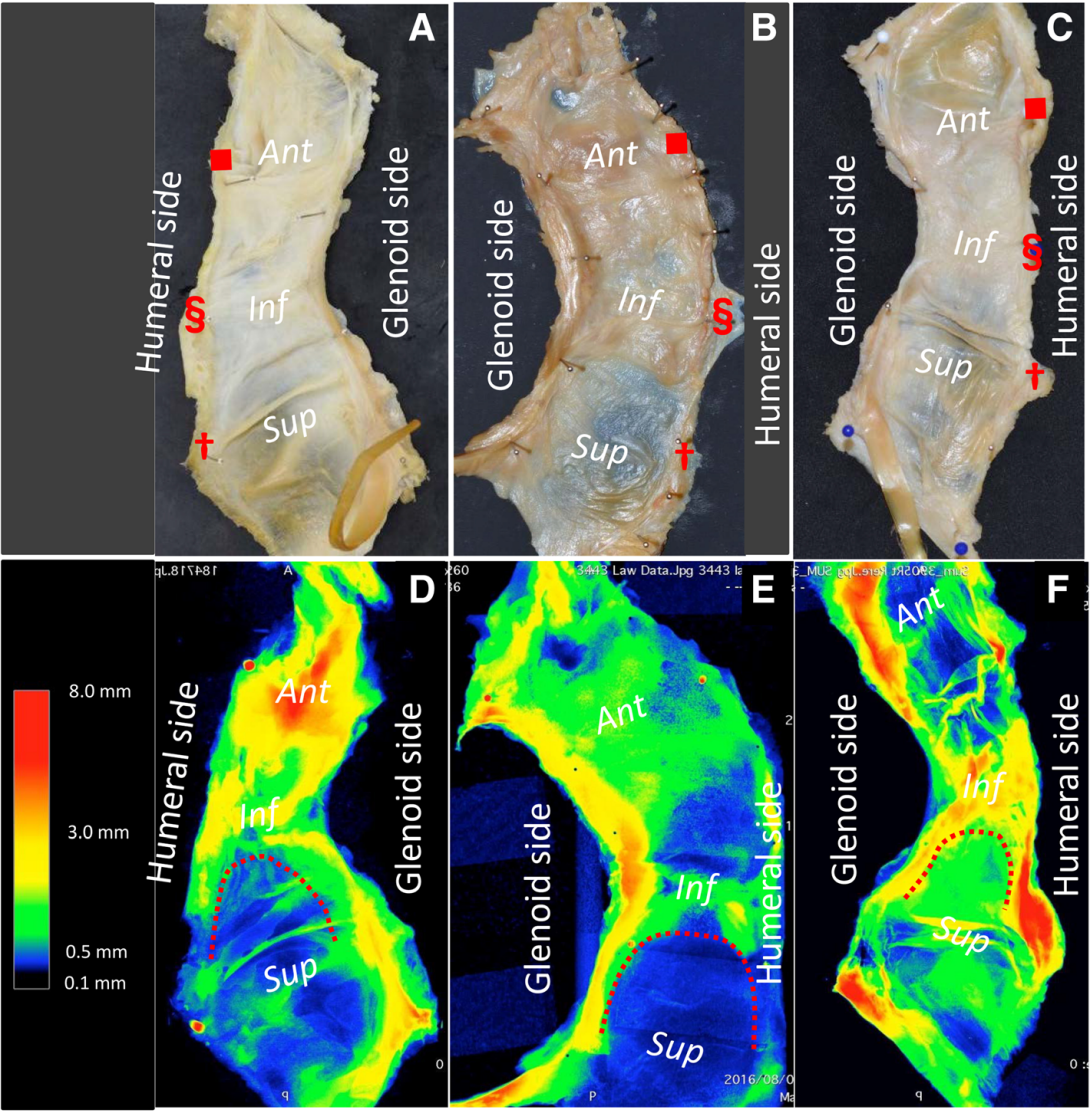
Variations in capsular thickness distribution. The remaining data, except for that in Fig. 6, is shown in the present figure. Inside appearances of the whole capsule (**a**, **b**, and **c**) corresponds to 3D images with micro-CT (**d**, **e**, and **f**), respectively. The color bar represents approximate thickness (mm) corresponding to the colors. Inferior parts of the capsule, colored red, yellow, and green, were consistently thicker than the superior part, colored green, blue, black. However, the anterior part seemed variable in comparison with the inferior part. In addition, the thick inferior part of the capsule continued to the superior area along the glenoid and humeral side edge (red dotted lines in **d**, **e**, and **f**). *Ant*, anterior; *Inf*, inferior; *Sup*, superior

The construct of the whole capsule demonstrated heterogeneous thickness in accordance with the location of bony attachments (Fig. 6). We divided the whole capsule into 3 parts: anterior, inferior, and superior, according to the humeral attachments (Fig. 6a). Inferior parts of the capsule, colored red, yellow, and green, were consistently thicker than the superior parts, colored green, blue, black (Fig. 7). However, the anterior part seemed variable in comparison with the other parts. In addition, the thick inferior part of the capsule continued to the superior area along the glenoid and humeral side edge (red dotted lines in Fig. 7).

## Discussion

Here we showed a comprehensive investigation of the insertion widths and capsular thickness of the whole glenohumeral articular capsule. From the inferior edge of the subscapularis insertion, via the humeral anatomical neck, to the inferior edge of the teres minor insertion, the inferior part of the articular capsule had a wider attachment on the humerus than other areas. In addition, the midsubstance of the articular capsule corresponding to the above-metioned area was thicker than the superior part of the capsule.

Previously, the superior capsule of the shoulder joint had been assumed to be a thin continuous sheet which was the deepest layer of rotator cuff tendons.(Clark and Harryman 2nd, 1992) Therefore, the functional significance of the superior capsule of shoulder joint were mostly overlooked before the following papers. Recently, Mihata et al.(Mihata et al. 2013; Mihata et al. 2012) reported the biomechanical significance and the good clinical results of the superior capsule reconstruction for irreparable rotator cuff tears. In addition, Nimura et al.(Nimura et al. 2012) recently reported that the articular capsule has a wide attachment on the humerus, at the border from the infraspinatus to the teres minor, and that it compensates for the lack of tendinous insertion. In addition, they described that it was consistent with the posterior pole that holds up the bridge cable(Burkhart 1993) or the posterosuperior glenohumeral ligament.(Pouliart et al. 2007) Additionally, the wide attachments of the joint capsule have been reported to be related with the stabilizing structures of the elbow and knee joints.(Nasu et al. 2017; Shimura et al. 2016) In the current study, we further demonstrated that the articular capsule, compared to other areas, had a wider attachment on the humerus from the inferior edge of the subscapularis insertion to the inferior edge of the teres minor insertion, where no rotator cuff tendons insert. The glenohumeral joint has been well known as the inherently unstable joint, and thought to be stabilized by the interaction between the static, including the labrum and capsule, and dynamic, including musculotendinous structures.(Johnson and Ellis 2005) Therefore, the attachment of the inferior capsule could be speculated to make up for the lack of rotator cuff insertion.

The inferior glenohumeral ligament complex (IGHL), which is a hammock-like structure of the articular capsule extending from anteroinferior to posteroinferior areas of the shoulder joint,(O'Brien et al. 1990) corresponds to the inferior part of the capsule as described in the current study. In previous reports, the dimensions of the IGHL attachment on the humerus have remained controversial. O’Brien et al.(O'Brien et al. 1990) and Ticker et al.(Ticker et al. 1996) reported that IGHL has 2 variable attachments on the humerus: a collar-like attachment close to the articular cartilage, and a V-shaped attachment pointing inferiorly. In addition, Sugalski et al.(Sugalski et al. 2005) also described 2 attachment variations: split-type and broad-type attachment on the humerus. To the contrary, Pouliart and Gagey(Pouliart and Gagey 2005) observed the IGHL attachment on the humerus by dissection and arthroscopic viewing, and reported that the IGHL consistently attaches to the humerus with a V-shape as viewed from outside, and it attaches close to the cartilage rim, when viewed from the intra-articular side. They explained that the discrepancy of the IGHL attachment findings compared to previous studies originates from these biases according to dissection method. The results of the present study were compatible with the description of Pouliart and Gagey. The current study showed that widths of the capsular attachment at the inferior edge of the subscapularis and teres minor insertion were relatively narrow, and they widely expanded toward the metaphysis of the humeral neck. Through the perspective of the whole capsular membrane, we could interpret that area of humerus as a wide attachment of the inferior part of the articular capsule in the continuous capsular membrane and its attachment, which corresponds to the capsuloligamentous complex such as IGHL.

The posterior band of the inferior glenohumeral ligament (PIGHL) has been described as the most significant stabilizer in the posterior loading position, however, the posterior capsule itself seemed relatively thin and the biomechanical performance is not robust unlike the thick anteroinferior capsule.(Bey et al. 2005; Ticker et al. 1996) In the current study, the red dotted line in Fig. 7 seems to correspond to the superior margin of PIGHL. Based on the current study, the humeral and glenoid side edges, which superiorly continued from the thick inferior part of the articular capsule could be interpreted as the PIGHL. Morphologically, the PIGHL could be speculated to act as the hammock suspension to superiorly pull in the thick inferior part of the articular capsule.

As for clinical implications, the detailed anatomic findings of the current study of the whole glenohumeral capsule could provide some clues to understand the significance of stabilizing techniques for shoulder instability. Some surgeons have proposed the importance of AIGHL tensioning for stabilization with lifting the AIGHL superiorward in the procedure of Bankart repair.(Blasier et al. 1992; Post 1996) In addition, more recent articles have indicated that the Hill-Sachs remplissage procedure together with the Bankart procedure provide satisfactory biomechanical stabilization and clinical outcomes.(Boileau et al. 2012; Cho et al. 2016; Merolla et al. 2015; Omi et al. 2014) The Bankart repair and remplissage have been thought to have no anatomic relationships, because both techniques affected to different parts of the glenohumeral capsule. In the current study, we revealed that the inferior part of the articular capsule is thicker than the superior part in the whole capsule, and the humeral and glenoid side edges continue superiorly. Bankart repair involves the anterior advance of the glenoid side in the thick inferior capsule. On the other hand, remplissage involves the superior advance of the humeral side of the thick inferior capsule as a continuous membrane (dagger in Fig. 6a). Based on the current study, these two techniques apply tension to the same structure but at different areas diagonally continued. Thus, these anatomic findings might support the efficacy of the Bankart repair combined with remplissage. However, the question whether the appropriate tension can be applied to the diagonally continuing capsule still remains. Next, based on the “circle stability concept”, the shoulder capsule has been thought to be damaged on both the posterior and anterior side to allow the complete dislocation. (Bowen and Warren 1991) Taking into consideration of this concept, the anatomical knowledge about the superior thinner part of the capsule could be some clue for understanding the pathomechanism of the anterior instability, differently from the IGHL. Based on the above, the anatomical findings in the current study could then serve as a take off point for further research regarding the pathology of the shoulder instability using the biomechanical and clinical studies.

The current study has several limitations. First, we used cadavers which were embalmed with formalin for measurements of the capsular attachment on bones. The position of the glenohumeral joint at the time of fixation may affect the local thickness of the whole capsule. However, the width of the capsular attachment should not be affected by the fixation methods or by the arm position. Additionally, Thiel’s embalming method was used for the analysis of capsular thickness to reduce the positional bias. The advantage of formalin-fixed specimens is thought to be that membranous structures can be clearly separated between the rotator cuff and capsule, as previously reported.(Nimura et al. 2012) Second, the current study was a pure anatomic study. Therefore, to show the significance of the thick inferior part of the articular capsule, biomechanical analyses and clinical findings are also recommended. Third, the results of the current study may be affected with the bias of age and female predominance. Fourth, regarding the thickness measurement of the whole capsule, we have not statistically analyzed the differences, and the method using micro-CT for the thickness measurement could not be validated by other methods, because we had no choice for non-contact measurement of the thickness. Fifth, the long head of the triceps muscle had the superficial fibers originating from the outer surface of the shoulder joint capsule. In the current study, the superficial fibers of the long head of the triceps muscle were cut at the base of the glenoidal origin. Therefore, the inferior part of the capsule could include the superficial fibers originating from the outer surface of the shoulder joint capsule.

## Conclusion

This anatomic study showed the wide humeral attachment of the articular capsule, in the area lacking rotator cuff insertion, from the inferior edge of the subscapularis insertion to the inferior edge of the teres minor insertion via the anatomical neck of the humerus. To comprehend the thick part of the whole capsule is important to perform the adequate surgery for the shoulder instability.
